# Decreased Ratio of Treg Cells to Th17 Cells Correlates with HBV DNA Suppression in Chronic Hepatitis B Patients Undergoing Entecavir Treatment

**DOI:** 10.1371/journal.pone.0013869

**Published:** 2010-11-08

**Authors:** Ji-Yuan Zhang, Chun-Hui Song, Feng Shi, Zheng Zhang, Jun-Liang Fu, Fu-Sheng Wang

**Affiliations:** 1 Research Center for Biological Therapy, Beijing 302 Hospital, Beijing, China; 2 Institute of Microbiology, Chinese Academy of Sciences, Beijing, China; New York University, United States of America

## Abstract

**Background:**

Treatment with nucleotide analogs is known to be effective in inhibiting HBV replication; however, patients with chronic hepatitis B (CHB) often show a wide range of clinical responses to these drugs. Therefore, the identification of an early immunologic marker associated with the clinical outcomes in such cases is critical for the improved clinical management. In our study, we aimed to investigate whether the viral load in CHB patients affected the ratio of the number of regulatory T cells (Tregs) to the number of interleukin-17-producing helper (Th17) cells. Further, we evaluated the clinical implications of the alterations in this ratio.

**Methodology/Principal Findings:**

Nine patients seropositive for hepatitis B e antigen received entecavir monotherapy for 12 months and the percentages of Tregs and Th17 cells as well as the HBV-specific IL-17 productions in these patients were longitudinally analyzed. The entecavir-induced suppression of HBV replication was accompanied by a rapid increase in the number of Th17 cells, together with a decrease in Treg cells, which lead to a significant reduction of Treg/Th17 ratios. In addition, peripheral blood mononuclear cells (PBMCs) exhibited a decreased IL-17 production upon stimulation with the HBV core antigen *in vitro*.

**Conclusions:**

The inhibition of viral replication results in an increase in Th17 cells and concomitant decrease in Treg cells. This imbalance of Treg cells to Th17 cells might have an important role in HBV persistence during entecavir treatment.

## Introduction

Patients who are chronically infected with hepatitis B virus (HBV) are at a risk of developing liver cirrhosis and hepatocellular carcinoma owing to many factors. One such factor is the host immune response, which is induced by viral persistence; it is related to the chronic inflammatory state of the patient and influences both the clinical outcome of HBV infection and clinical antiviral efficacy [1]–[3]. Antiviral treatment with nucleos(t)ide analogs is one of the therapeutic options for patients with chronic hepatitis B (CHB). These drugs effectively suppress HBV replication, alleviate liver injury, and decelerate disease progression. However, CHB patients often exhibit varied responses to these drugs [4], [5]. Therefore, the identification of an early immunologic marker associated with the clinical outcomes of the antiviral therapy is critical for the development of improved clinical management and therapeutic options.

Helper CD4^+^ T cells can orchestrate host immune responses through the release of distinct cytokine profiles. Recent studies have described 2 additional subsets—regulatory T cells (Tregs) and interleukin (IL)-17-producing CD4^+^ T helper (Th17) cells [6], [7]—and have shown the reciprocal relationships between T helper subsets and the outcome to anti-HBV therapy [8], [9]. Recent studies have shown that CHB patients have increased percentages of Tregs in their peripheral blood and liver [10]–[13]. The increased levels of Tregs, which is associated with increased HBV-DNA levels, can inhibit the HBV-specific immune response in a dose-dependent manner, and the depletion of Tregs results in increased HBV-specific CD4^+^ and CD8^+^ T-cell proliferation and IFN-γ production. Adefovir-induced viral load reduction lead to the decline in the number of circulating Tregs together with a partial recovery of the immune response [13], [14]. These findings, therefore, suggested that Tregs actively participate in regulating anti-HBV response. Furthermore, Th17 cells are significantly increased in CHB patients and are considered to be important factors triggering liver damage [15]. However, to date, the role of Th17 cells during antiviral therapy remain unknown. Tregs and Th17 cells are closely associated with each other, and tumor growth factor (TGF)-β orchestrates the differentiation of both these cells in a concentration-dependent manner [16]–[18]. Low concentrations of TGF-β synergize with IL-6 and IL-21 to promote IL-23R expression, thereby favoring Th17 cell differentiation. However, increased concentrations of TGF-β repress IL-23R expression and favor the generation of Tregs. Considering all these evidences together, we hypothesize that an imbalance of Th17 cells and Tregs may exist and may play a role in regulating the immune response during anti-HBV therapy.

Entecavir is a carbocyclic analog of 2′-deoxyguanosine, and it is more potent than lamivudine and adefovir against HBV replication. Currently, entecavir is used as the first-line anti-HBV therapy [19]–[22]. In our study, we aimed to investigate the influence of the decrease in the viral load on the percentages of Tregs and Th17 cells, and thereby assess the effectiveness of antiviral therapy in CHB patients.

## Methods

### Patients

Twenty-eight treatment-naive patients with CHB who visited the Beijing 302 Hospital (Beijing, China) were enrolled in this study. All patients were seropositive for hepatitis B surface antigen (HBsAg) and hepatitis e surface antigen (HBeAg), in which nine patients were followed serially with protocol visits for 12 months continuously during the course of the antiviral treatment with a nucleoside analogue (entecavir, 0.5 mg/day). They were 6 men and 3 women (mean age, 33.3±5.2 years) without concurrent HCV, hepatitis G virus, hepatitis D virus, HIV-1 infections and autoimmune liver diseases. At baseline, their HBV-DNA levels were >2×10^4^ IU/ml (range, 7.17×10^5^–8.34×10^8^ IU/ml) and alanine aminotransferase (ALT) levels were twice (range, 83–369 U/L) the upper limit of the normal levels. Twenty-six healthy individuals were enrolled as normal control (HC). The study protocol was approved by Beijing 302 Hospital Research Ethnics Committee, written informed consents for therapy and study was obtained from each patient. The clinical characteristics of these subjects are listed in Table 1.

**Table 1:** Clinical characteristics of the populations enrolled in the study.

Group	HC	CHB
Case	26	28
Sex (male/female)	16/10	18/10
Age (years)	31 (20–44)	30 (18–46)
ALT (U/L)	21 (10–37)	119 (81–443) *
HBV DNA (IU/ml)	ND	3.20×107(2.57×105–2.01×109) *

Data are shown as median and range. HC: healthy control; CHB: chronic hepatitis B; ND: not determined.

*P<0.05 vs. CHB subjects.

### Flow cytometric analysis

All antibodies were purchased from BD Biosciences (San Jose, CA), except for phycoerythrin (PE)-conjugated anti-IL-17A and fluorescein isothiocyanate (FITC)-conjugated anti-FoxP3 from eBioscience (San Diego, CA). For immunostaining of intracellular IL-17A, fresh heparinized peripheral blood (200 µl) was incubated with phorbol-12-myristate-13-acetate (PMA, 300 ng/ml, Sigma-Aldrich, St. Louis, MO) and ionomycin (1 µg/ml, Sigma-Aldrich) in 800 µl of RPMI 1640 medium supplemented with 10% fetal calf serum for 6 hours. Monensin (0.4 µM, BD PharMingen, San Diego, CA) was added during the first hour of incubation. The blood cells were then lysed with FACS™ lysing solution (BD PharMingen), further permeabilized and stained with the corresponding intracellular antibody. For intracellular FoxP3 staining, peripheral blood (200 µL) was firstly lysed with FACS™ lysing solution (BD PharMingen), and then treated with eBioscience fix/perm (eBiosciences) according to the manufacturer's instructions. Cells were incubated with FITC-conjugated anti-FoxP3 for 30 min, fixed, and analyzed using FACSCalibur (BD Biosciences) and FlowJo software (Tristar, USA) by following the protocol described in a previous study [15].

### Cell stimulation

Freshly isolated peripheral blood mononuclear cells (PBMCs) were stimulated with HBcAg as described in a previous study [15]. Briefly, PBMCs were cultured in triplicate in 96-well plates at a concentration of 5×10^5^ cells/well in 200 µl of RPMI 1640 containing 10% fetal calf serum. The cells were stimulated with HBcAg (5 µg/ml, ARP, Belmont, MA) and cultured for 3 days. After 3 days of incubation, the supernatants were collected for the measurement of serum IL-17 levels.

### Enzyme-linked immunosorbent assay

The levels of IL-17 in the plasma and supernatants were measured using quantitative sandwich enzyme-linked immunosorbent assay (ELISA) by following the manufacturer's instructions (R&D Systems, Minneapolis, MN). The minimal detectable concentration of IL-17 was 15 pg/ml. The intra-assay and inter-assay coefficients of variation for all the ELISAs were <5% and <10%, respectively. All samples were measured in duplicate.

### Virological and immunological assessment

The levels of HBsAg, anti-HBs, anti-HBc, HBeAg, anti-HBe, anti-HCV, anti-HDV, anti-HGV, and anti-HIV were measured using commercially available kits (Abbot Laboratories, North Chicago, IL). HBV-DNA levels were determined using real-time polymerase chain reaction (Amplicor, Roche) according to the manufacturer's instructions. The threshold of the HBV DNA detection limit was 100 IU/ml [23].

### Statistical Analysis

All data were analyzed using SPSS software (SPSS Inc., Chicago, IL). For comparison between different time points, statistical comparison was analyzed using the Wilcoxon matched pairs test. The correlations between the variables were evaluated using the Spearman rank correlation test. For all tests, two-sided *P* values less than 0.05 were considered significant.

## Results

### Treg/Th17 ratios were decreased in CHB patients compared with HCs

**Figure 1:**
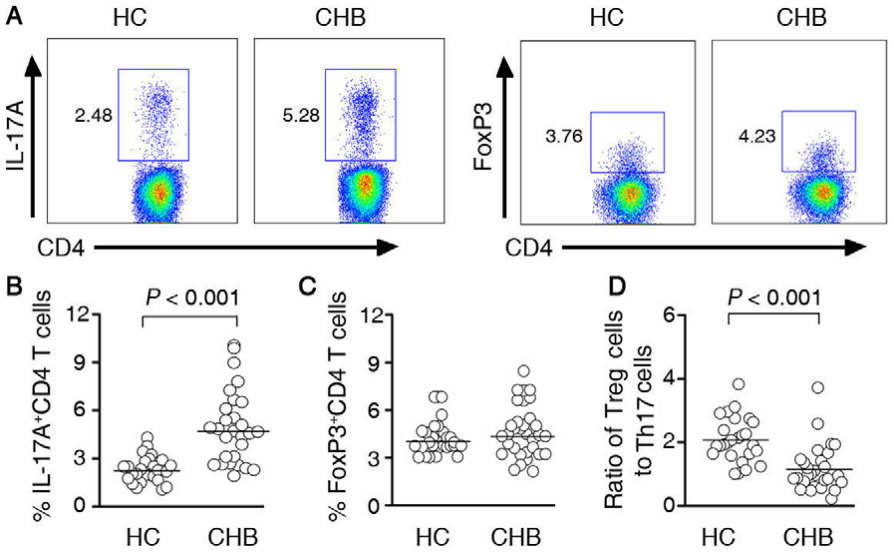
The Treg/Th17 ratio was decreased in CHB patients. (A) Representative dot plots of IL-17A and FoxP3 expression in peripheral CD4^+^ T cells of HC subjects and CHB patients. The values in the quadrants indicate the percentage of each CD4^+^ T-cell subset. (B) Pooled data indicate the percentages of Th17 cells in HC and CHB patients. (C) Pooled data indicate the percentages of Treg cells in HC and CHB patients. (D) The ratio of circulating Treg cells to Th17 cells is significantly lower in CHB patients. Horizontal bars represent the median values of indicated index.

We measured the frequency of IL-17A-producing cells (Th17) and FoxP3-positive cells (Tregs) within the CD4 subset using flow cytometry. All subjects clearly displayed the two CD4 T-cell subsets (Figure 1A). We showed a significantly higher frequency of Th17 cells in CHB patients (5.16%±2.21) when compared with healthy subjects (2.04%±0.56, p<0.001) (Figure 1B). Despite Tregs can generate from the same naïve T cell poll that generates Th17 cells, we found no significant differences in Treg frequencies when comparing CHB patients with HCs (Figure 1C). To tie Treg cells with Th17 cells, we use the ratio of Treg cells to Th17 cells as an index; we observed a significantly lower ratio in CHB patients (Figure 1D).

### HBV-DNA levels decreased and alanine aminotransferase levels normalized during entecavir treatment

**Figure 2:**
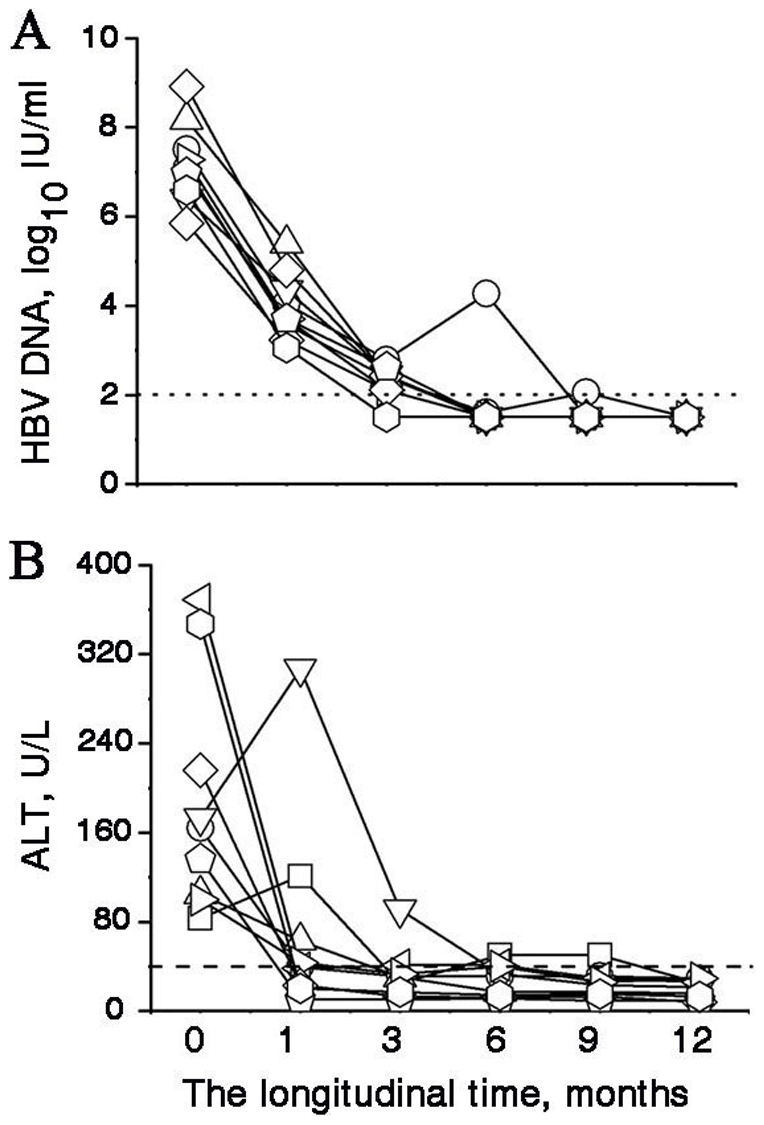
Clinical characteristics of chronic HBV patients undergoing entecavir therapy: (A) the serum HBV-DNA levels of the 9 patients at the baseline and during the 12 months of entecavir therapy; and (B) the serum ALT levels of the 9 patients at the baseline and during the 12 months of entecavir therapy. The levels of HBV DNA and ALT at month 1 were significantly lower than those at the baseline (*P*<0.05). Each symbol represents an individual and each line represents changes in an individual patient's DNA or ALT levels from the baseline to the endpoint (month 12). ALT, alanine aminotransferase; HBV, hepatitis B virus.

For entecavir-treated patients, the levels of serum HBV DNA and alanine aminotransferase level (ALT) were determined at the baseline, as well as at 1, 3, 6, 9, and 12 months after entecavir treatment. In all patients, the HBV replication at month 1 was considerably lower than that at baseline; the median reduction of HBV DNA level was 3.2 log_10_ IU/mL, which gradually reduced to 2.3 log_10_ IU/mL at 3 months, and this decrease was paralleled by a decrease in the serum ALT levels. At 6 months of treatment, HBV DNA was undetectable in 8 patients (89%) and ALT levels were normalized in 7 (78%) patients. At the end of the follow-up, loss of serum HBeAg was achieved in 7 patients, and seroconversion from HBeAg to anti-HBeAg was achieved in 2 patients. Loss of serum HBsAg during the treatment was not achieved in any patient. The changes in the levels of clinical markers in these subjects are listed in Figure 2.

### Serial fluctuations of peripheral blood T helper subsets during entecavir-induced viral load reduction

**Figure 3:**
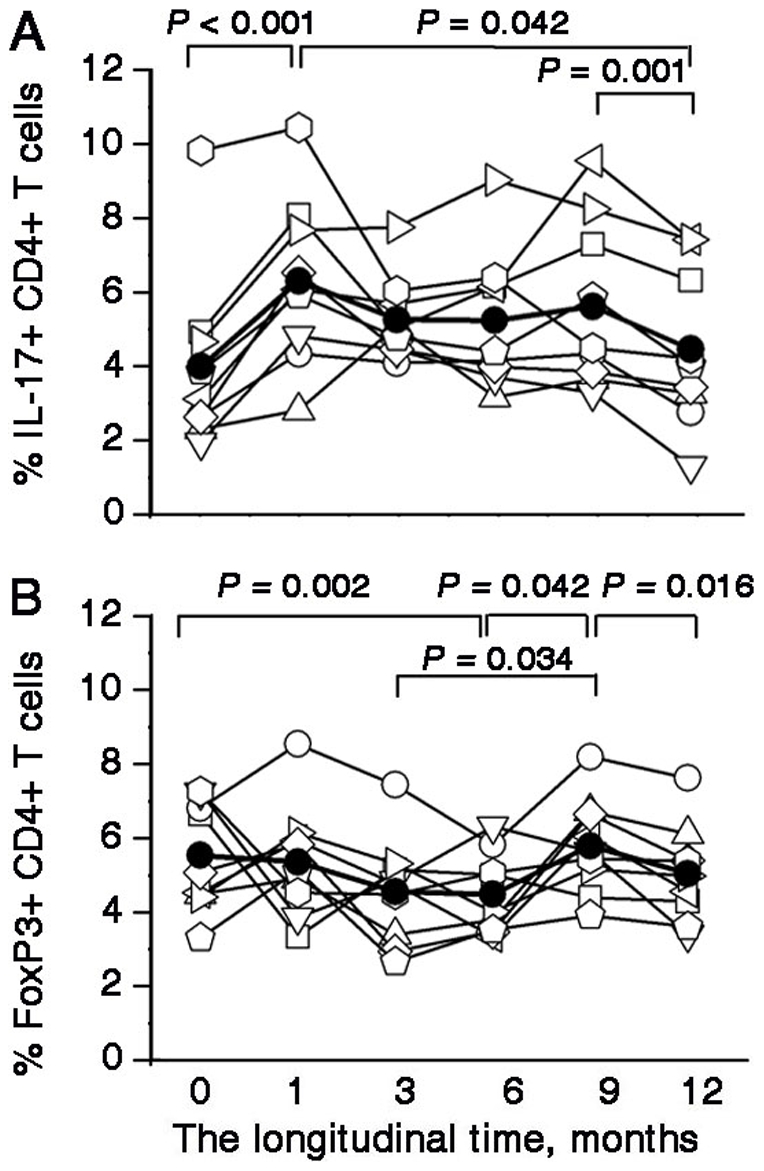
The frequencies of Th17 cells and Tregs in entecavir-treated patients during the course of therapy: (A) The frequencies of Th17 cells were observed from the baseline to the endpoint (month 12). The total number of Th17 cells was rapidly increased at month 1 and were higher than the baseline from month 1 to month 9; subsequently, their levels declined to the baseline levels at approximately the 12^th^ month of therapy. The *P* values between different time points are shown. (B) The frequencies of Th17 cells were observed from the baseline to the endpoint (month 12). The *P* values between different time points are shown. (A and B) Each symbol represents one individual and each line represents the changes in an individual patient's Th17 frequencies or Treg frequencies from the baseline to the endpoint (month 12). The solid line depicts the mean percentages of Th17 cells and Tregs during the antiviral therapy.

To assess the effect of entecavir treatment on T-helper subsets, we longitudinally determined the proportion of Th17 cells and Tregs in the blood samples of these patients by using the flow cytometric assay. All patients showed distinct compositions of CD4^+^ T-cell subsets at different treatment time points. Interestingly, the frequencies of Th17 cells reached the maximal peak at month 1 and the frequencies at all time points from month 1 to month 9 remained higher than those at the baseline and at the endpoint (Figure 3A). In contrast, the frequencies of Tregs gradually decreased from the baseline to month 6 and subsequently, they showed a reverse “V”-type change with the maximal peak at month 9 (Figure 3B).

**Figure 4:**
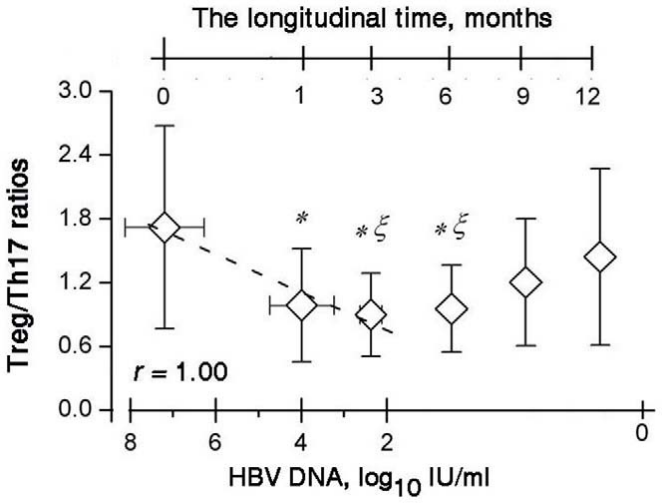
Correlations between Treg/Th17 ratios and HBV-DNA levels. The average Treg/Th17 ratios and HBV-DNA levels for each time point are represented by the diamond symbols. The reduction in the Treg/Th17 ratios from the baseline to month 3 correlates with the reduction in the HBV-DNA levels. Error bars illustrate the standard errors. * *P* = 0.039 for month 1, *P* = 0.016 for month 3, *P* = 0.006 for month 6 compared with the baseline. § *P* = 0.038 for month 3, *P* = 0.015 for month 6 compared with the baseline.

Tregs and Th17 cells may be generated from the same precursor T cells. Therefore, we investigated the reciprocal relationship between Tregs and Th17 cells during the treatment. As shown in Figure 4, the Treg/Th17 ratios were the lowest at month 3 and exhibited a reverse “V”-type change. We further dissected the association between the Treg/Th17 ratios and clinical implications in these CHB patients and found that the Treg/Th17 ratios were positively correlated with HBV-DNA levels during the period from the baseline to month 3.

### IL-17 production decreased during entecavir-induced viral load reduction

**Figure 5:**
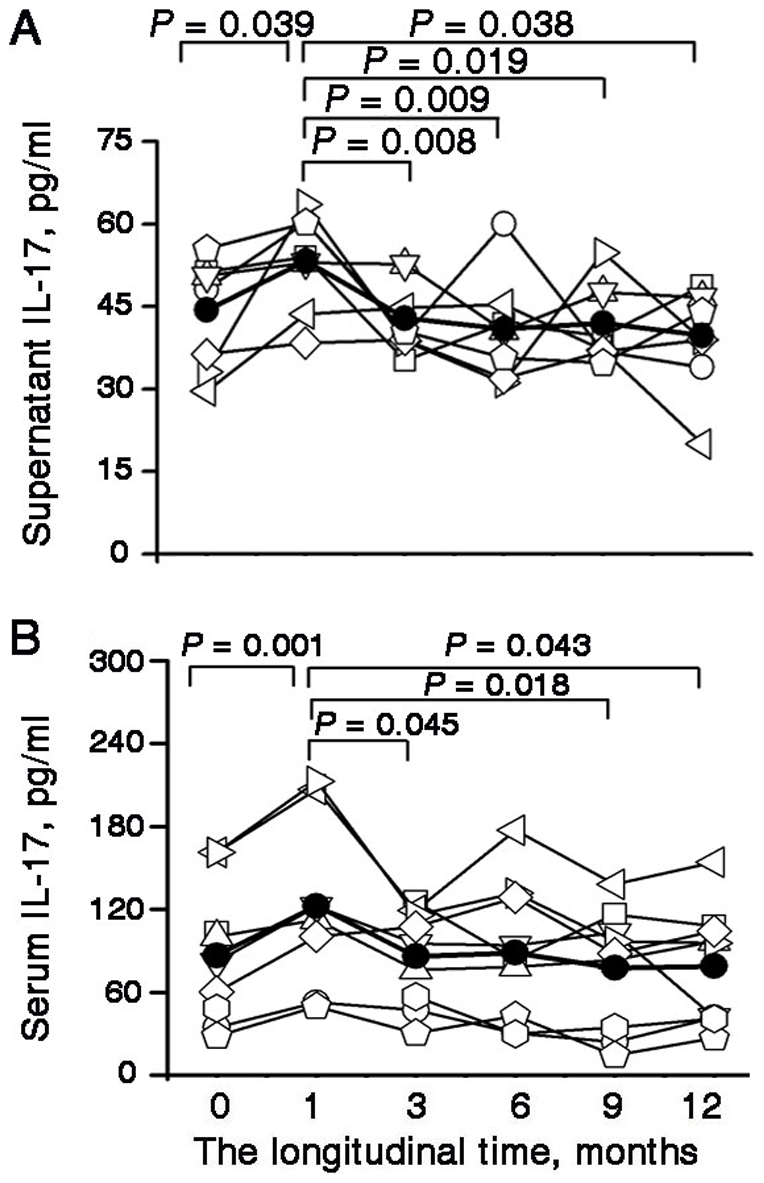
IL-17 production by PBMCs and IL-17 concentrations in serum (A) PBMCs, obtained at baseline and during the 12 months of entecavir therapy, were stimulated for 3 days with HBcAg. The IL-17 level in the supernatants was determined using ELISA. The *P* values between the different time points are shown. (B) Serum IL-17 concentrations at different time points were detected using ELISA. The *P* values between different time points are shown. (A and B) Each symbol represents one individual and each line represents the changes in an individual patient's IL-17 concentrations in the supernatants or IL-17 concentrations in the serum from the baseline to the endpoint (month 12). The solid line depicts the mean IL-17 concentrations in the supernatant or serum. PBMCs: peripheral blood mononuclear cells; IL- interleukin; ELISA: enzyme-linked immmunosorbent assay.

To determine the levels of HBV-specific IL-17, PBMCs collected at the baseline and during therapy were stimulated with HBcAg. The HBcAg-specific IL-17 production at month 1 was higher than that at the baseline, which was consistent with the total frequencies of IL-17-producing cells, as determined by intracellular staining. However, the HBcAg-specific IL-17 production gradually decreased from month 3 to the end of the follow-up period. Therefore, the HBcAg-specific IL-17 productions showed a reverse “V”-type change with the maximal peak at month 1 (Figure 5A); the serum IL-17 levels, as determined by ELISA, also showed a similar trend during entecavir treatment (Figure 5B).

## Discussion

The advantage of our study design is that by longitudinally monitoring the impact of treatment-induced suppression of HBV replication with direct antivirals (entecavir), we were able to define the cause-effect relationship between the viral load and Treg/Th17 ratio. Our findings showed that the Treg/Th17 ratio was decreased in CHB patients. In addition, entecavir-induced suppression of viral replication resulted in an increase in Th17 cells and concomitant decrease in Treg cells, which was manifested by a profound decrease of the Treg/Th17 ratios. Thus, this imbalance of Treg cells to Th17 cells might play an important role in HBV persistence during entecavir treatment.

Increasing evidences have shown that the host immunity influences antiviral efficacy in CHB patients. We believed that restoration of HBV-specific T-cell response during antiviral therapy is associated with CD4^+^ T-cell activity. For example, lamivudine was found to increase the CD4^+^ T-cell response at the initial 1–2 weeks of antiviral therapy [24]. Furthermore, the combination therapy of adefovir dipivoxil and emtricitabine led to an increase in the CD4^+^ T-cell activity [25]. Previous study has shown that adefovir treatment can partially reduce the frequency of the circulating Tregs and that this reduction occurs concomitantly with an increase in the HBV-specific T cell response [13], [14]. In our study, we also found that entecavir treatment could partially reduce the frequency of circulating Tregs within 6 months. Th17 cells have been associated with the immunopathological changes in several chronic inflammatory diseases and could conceivably play a major role in the inflammatory state associated with liver disease [15], [26]–[29]. Therefore, we hypothesized that the number of Th17 cells may decrease during anti-HBV therapy. Unexpectedly, we found that the total number of Th17 cells was indeed increased from month 1 and the total number of these cells throughout the period from month 1 to month 9 was higher than that at the baseline; subsequently, their levels declined to the baseline levels at approximately 12 months of therapy. Given the mechanism of action of entecavir, which directly blocks viral replication, it seems likely that therapy results in a siginificant fall in the HBV-DNA levels and that this in turn leads to a change in the cytokine environment, which, on one hand, favor the increase in the numbers of Th17 cells, on the other hand, may lead to a decrease in Tregs. This is similar to what has been reported in autoimmune disease [30]. Interestingly, there was a temporary increased HBV-specific IL-17 production at month 1, which occurs concomitantly with the decrease in the numbers of Tregs. However, the temporary increased HBV-specific IL-17 production gradually declined from month 1 to the baseline level. Together with our recent study in which we observed that the response of Th17 cells was influenced by the inflammatory environment (unpublished data), future studies should be undertaken to elucidate the factors that suppress the activity of Th17 cells during anti-HBV therapy.

Recent reports have shown that Treg/Th17 ratio may be a useful marker for assessing the severity of diseases in animal models and human diseases, and important mechanisms were postulated to explain the skewed Treg/Th17 ratios [31], [32]. However, no study has described the significance of the skewed Treg/Th17 ratios during antiviral therapy in CHB patients. In this study, we highlighted the existing direct relationship between Treg/Th17 ratios and HBV-DNA levels. We observed that Treg/Th17 ratios tended to decrease during the first 3 months of treatment in all patients. Interestingly, the Treg/Th17 ratios seem to increase after HBeAg loss. HBeAg was considered to be a tolerogen in HBV infection. However, the limited sample population of entecavir-treated patients in our study precluded us from testing the relationship between HBeAg and Treg/Th17 ratios. We will further investigate the Treg/Th17 ratios before and after seroconversion in the absence of changes in the HBV-DNA levels.

In conclusion, this study highlights that the viral load affects the Treg/Th17 ratios during antiviral treatment in CHB patients. Our findings reveal a strong correlation between HBV load and the Treg/Th17 ratios during antiviral treatment and these data indicated the imbalance of Treg cells to Th17 cells might play an important role in HBV persistence.
